# CT features and outcomes of newly developed pulmonary lesions in patients with Coronavirus Disease 2019 (COVID-19)

**DOI:** 10.7150/ijms.47587

**Published:** 2020-08-29

**Authors:** Wang Jia Li, Zhuo Ma Lv, Fa Jin Lv, BinJie Fu, Liang Bo Hu, Zhi Gang Chu

**Affiliations:** 1 Department of Radiology, The First Affiliated Hospital of Chongqing Medical University, Chongqing, China, 40016.; 2 Department of Radiology, Yongchuan Hospital of Chongqing Medical University, Chongqing, China, 40016.

**Keywords:** Coronavirus Disease 2019, Pneumonia, New lesions, Primary lesions, CT

## Abstract

**Background**: In patients with coronavirus disease 2019 (COVID-19) pneumonia, whether new pulmonary lesions will continue to develop after treatment was unknown. This study aimed to determine whether new pulmonary lesions will develop after treatment in patients with COVID-19 pneumonia, and investigate their CT features and outcomes.

**Methods:** This retrospective study included 56 consecutive patients with confirmed COVID-19 pneumonia from January 20 to March 5, 2020. Their initial and follow-up CT images and clinical data were reviewed. The CT manifestations of primary and newly developed pulmonary lesions and their changes after treatment were mainly evaluated.

**Results:** Among the 56 patients (mean age: 48±15 years, 35 men) with COVID-19 pneumonia, 42 (75.0%) patients developed new pulmonary lesions during treatment. All new lesions developed before the nucleic acid test turned negative. Patients with new lesions were more likely to have lymphopenia (*P*=0.041) or increased C-reactive protein (CRP) levels (*P<*0.001) than those without new lesions. Of the 42 patients, 30 (71.4%) patients developed new lesions once, and 12 (28.6%) twice or thrice, which usually appeared when primary lesions were progressing (37, 88.1%) and 1-15 days after treatment. The newly developed lesions were usually multiple (38, 90.5%), distributed in the previously involved (39, 92.9%) or uninvolved (27, 64.3%) lobes, and manifested as ground-glass opacities (GGOs) with consolidation (23, 54.8%) or pure GGOs (19, 45.2%). After their occurrence, the new lesions in most patients (32, 76.2%) showed direct absorption, whereas those in some patients (10, 23.8%) progressed before absorption.

**Conclusion:** During treatment, most patients with COVID-19 pneumonia will develop new pulmonary lesions, which usually manifest as multiple GGOs distributed around the primary lesions or in previously uninvolved lobes, and are subsequently absorbed directly.

## Introduction

Coronavirus disease 2019 (COVID-19) is a global pandemic caused by severe acute respiratory syndrome coronavirus 2 (SARS-CoV-2) 1, 2. Although SARS-CoV-2 has at least 70% similarity in genetic sequence to SARS-CoV 3, COVID-19 spreads much more rapidly than SARS 4. This might be because the initial symptoms of patients with COVID-19 pneumonia are not obvious and nonspecific, and asymptomatic carriers can transmit SARS-CoV-2 to other uninfected people, causing the virus to spread rapidly.

SARS-CoV-2 can be transmitted to others through droplets from infected patients 5. Also, the virus could be transmitted from primary lesions to the infected or uninfected lung tissue in patients with COVID-19 pneumonia. COVID-19 testing is performed by using nucleic acid tests of SARS-CoV-2, which remains the reference standard for diagnosing the disease 6. Multiple viral nucleic acid tests are used to determine whether patients still carry the virus and are contagious after treatment. However, it is unclear whether these tests can evaluate the patients' self-transmission accurately.

Since nucleic acid test can result in false-negative outcomes, chest CT plays a vital role in early diagnosis and follow-up of COVID-19 7, 8. The imaging characteristics and evolution rules of pulmonary lesions revealed in previous studies provided useful information for understanding this disease 9-13. However, they did not mention the newly developed lesions and analyze their CT features and outcomes. Therefore, this study aimed to determine whether patients with COVID-19 pneumonia would develop new lesions after treatment, and their related clinical factors, and to investigate their CT characteristics and following changes for understanding self-transmission better.

## Materials and Methods

This retrospective study used unidentifiable patient data and had no potential risk to patients. Therefore, the institutional review board of our hospital approved this study and waived informed consent.

### Study population

**Figure 1:**
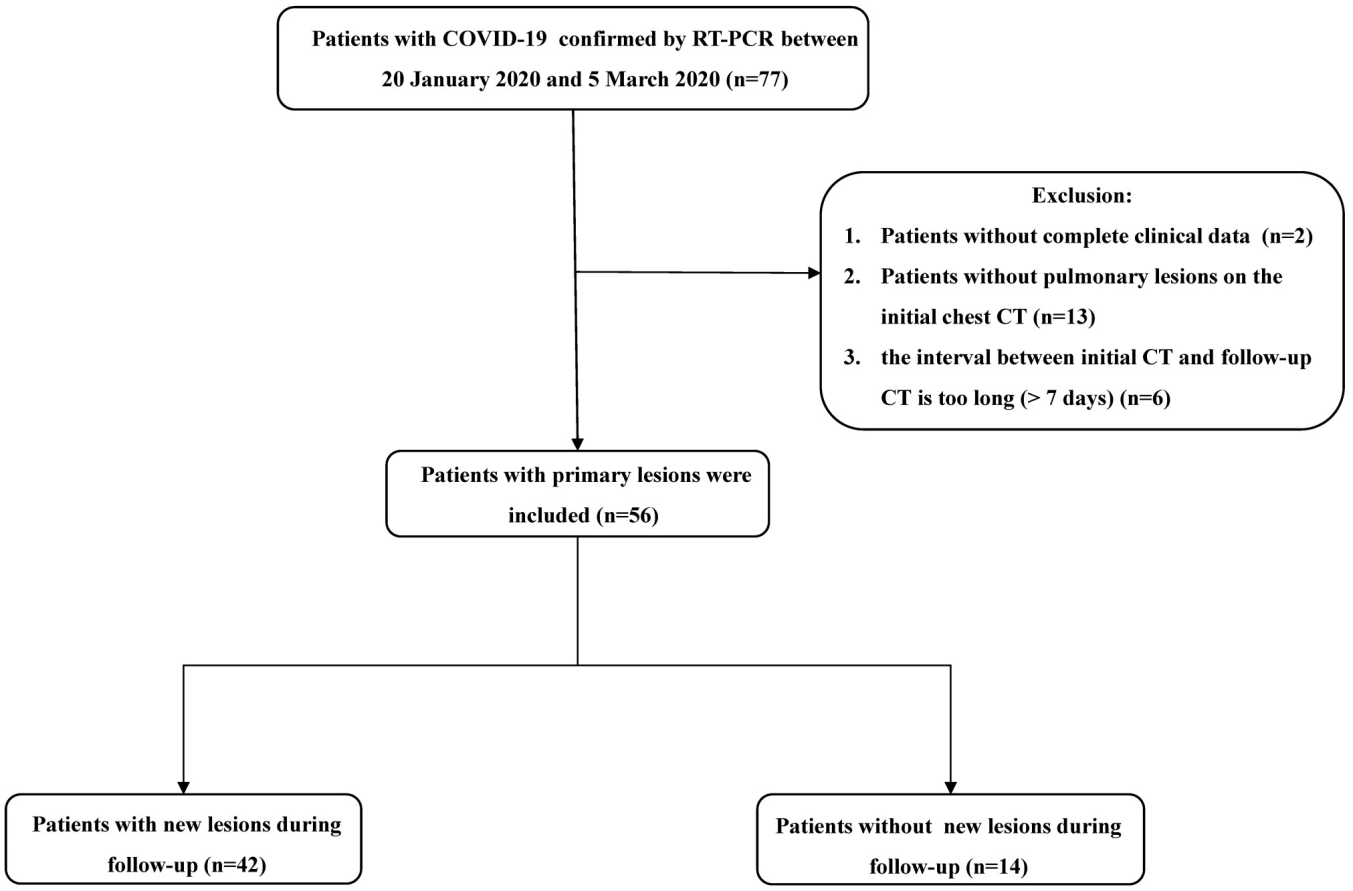
** Flowchart of study population.** Abbreviations: COVID-19: coronavirus disease 2019; GGOs: ground-glass opacities.

From January 20 to March 5, 2020, patients with confirmed COVID-19 were enrolled in this study. Upon admission, all patients underwent chest CT examinations. Follow-up CT scans were obtained during treatment. Two patients without complete clinical data, 13 patients without pulmonary lesions on initial CT, and six patients with the interval between two consecutive CT scans of > 7 days were excluded from the study. The flowchart of study population is shown in **Figure 1.**

### CT examinations

All patients underwent CT examinations upon admission to our hospital. Repeat follow-up CT scans for each patient were performed. All CT examinations were performed using two multi-detector CT scanners (Philips Brilliance iCT; Philips Healthcare, Massachusetts, United States, and Siemens SOMATOM go.Top; Siemens Healthineers, Erlangen, Germany) with the following parameters: 120 kV, automatic tube current modulation; detector collimation, 0.625/0.6 mm; rotation time, 0.5 s; pitch, 0.758/1.5; matrix: 512 × 512; section thickness and interval, 5.0 and 5.0 mm, respectively; and reconstructed thickness, 1 mm. All non-contrast CT scans were acquired with patients in a supine position and the patients were scanned from the thoracic inlet to the lung base at the end of inspiration.

### Analysis of CT characteristics

All CT data were analyzed on the picture archiving and communication system with a lung window setting (width, 1500 HU; level, -500 HU). Two senior radiologists (Z.G.C and F.J.L with 10 and 20 years of experience in thoracic imaging, respectively), who were blinded to the clinical data, independently evaluated the initial and follow-up CT images. The discrepancies were resolved by consensus.

For each patient, the distribution, extent, and CT features of the primary and newly developed lesions were evaluated. On follow-up CT images, changes in the primary and new lesions were evaluated. The new lesions were evaluated in the following aspects: (a) interval of initial CT to the first repeat CT with newly developed lesions, (b) number (single or multiple); (c) distribution (in previously involved lobes, in previously uninvolved lobes, or both); (d) density (pure GGO, GGOs with consolidation, or both), (e) shape (small patchy, nodular, or both), and (f) their changes during treatment (directly absorption or progression before absorption). Based on the presence or absence of newly developed lesions during treatment, the patients were divided into two groups: cases with new lesions and cases without new lesions.

### Clinical characteristics

Each patients' clinical and laboratory data were recorded by one radiologist (W.J.L). The clinical data included age, gender, clinical type, and initial symptoms. Laboratory findings, such as white blood cell, lymphocyte, C-reactive protein (CRP), lactate dehydrogenase (LDH), and partial pressure of oxygen (PaO_2_), were recorded. These laboratory tests were performed when the patients admitted to the hospital. Also, the frequency and results of nucleic acid tests were recorded.

### Statistical analysis

All data were statistically analyzed using Statistical Packages for the Social Sciences, version 20.0 (IBM Corp., Armonk, New York, USA). Data were expressed as mean ± standard deviation for continuous variables, and presented as numbers and percentages for categorical variables. The analysis of variance or Wilcoxon rank-sum test was used for continuous variables, and categorical variables were analyzed using the Pearson χ^2^ test or Fisher exact test. A P value of ≤ 0.05 was considered statistically significant.

## Results

Fifty-six patients, including 35 (62.5%) men (mean age, 48±15 years; range, 14-89 years) and 21 (37.5%) women (mean age, 52±13 years; range, 26-83 years), with a mean age of 48±15 years (range, 14-89 years), were enrolled in this study. Of the 56 patients, 42 (75.0%) developed new lesions during treatment. The patients' clinical and laboratory data are shown in **Table 1.** The mean duration from illness onset to admission was three days. Fever (38/56, 67.9%) and cough (38/56, 67.9%) were the most common symptoms. More patients with new lesions showed lymphopenia and increased CRP than those without (*P*=0.041 and *P<*0.001, respectively). All patients were confirmed to have a positive result for COVID-19 from nucleic acid tests of respiratory secretions upon admission and turned negative (twice in succession) before discharged. Besides, the nucleic acid tests of SARS-CoV-2 in all patients with new lesions turned negative after all new lesions developed.

### CT imaging findings

Of the 56 patients, primary pulmonary lesions were usually multiple (53, 94.6%), frequently involved multiple lobes (38, 67.9%), mainly distributed in the lower lobes (47, 83.9%), and manifested as pure GGOs (33, 58.9 %) and GGOs with consolidation (23, 41.1%). The involved lobes (3.5±1.5 vs. 3.1±1.4, *P*=0.402) and segments (7.9±5.0 vs. 7.2±1.4, *P*=0.489) in patients with and without new lesions were similar.

**Table 2** presents the CT features of newly developed pulmonary lesions in patients with COVID-19. Among 42 patients with new lesions, 23 (54.8%) developed new lesions 1-4 days after the initial CT. New lesions developed once in 30 (71.4%) cases, and twice and three times in 12 (28.6%) cases. They were usually multiple (38/42, 90.5%), distributed around primary lesions (39/42, 92.9%) or in the previously uninvolved lobes (27/42, 64.3%), and manifested as GGOs with consolidation (23/42, 54.8%) or pure GGOs (19/42, 45.2%). The number of new lesions developed in different periods was similar (*P*=0.377), with a mean number of 6.2 ± 4.8.

### Changes of primary and newly developed lesions during follow-up

**Figure 2:**
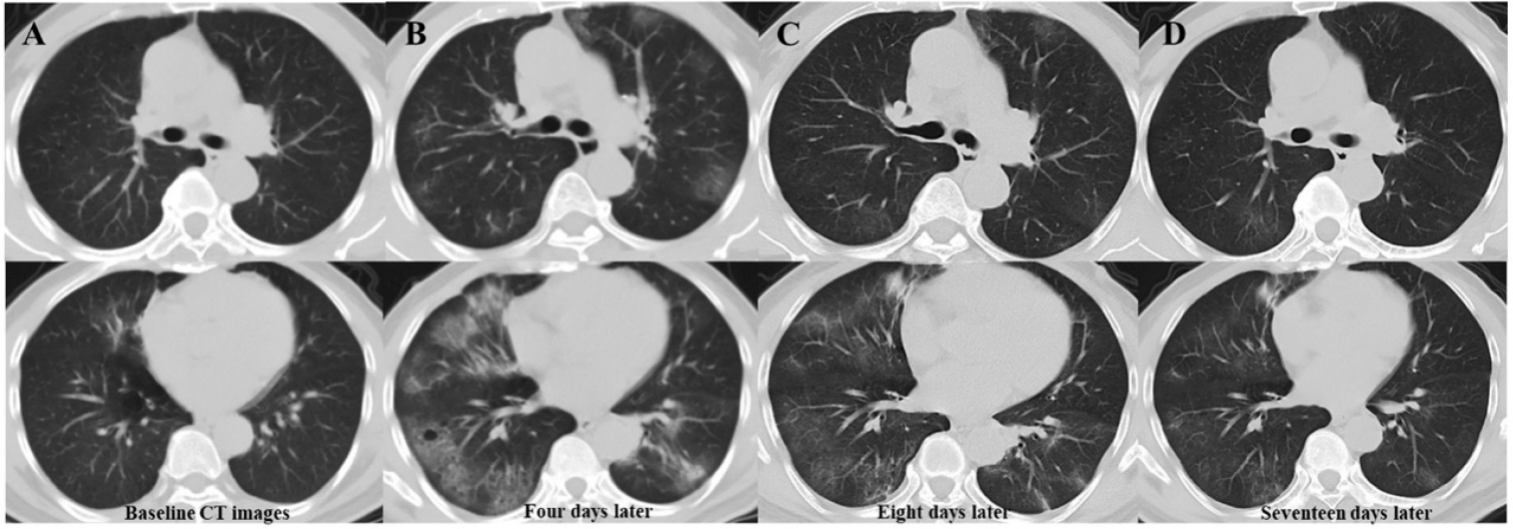
** Serial chest CT images of a 63-year-old man with confirmed COVID-19 pneumonia.** Baseline CT images (column A) show patchy ground-glass opacity (GGO) in the right middle lobe. Four days later (column B), the primary lesion progress, and multiple new GGOs occur in previously involved and uninvolved lobes. Follow-up CT images (column C) eight days after admission show absorption of the primary and newly developed lesions. The latest follow-up CT images (column D) 17 days after admission show significant absorption of lesions, with little residue. Abbreviations: COVID-19: coronavirus disease 2019; GGOs: ground-glass opacities.

**Table 3** presents follow-up CT characteristics of patients with COVID-19. During treatment, primary lesion progression was significantly more common in patients with new lesions than in those without. (90.5% vs. 14.3%, *P*=0.000). Most patients (37/42, 88.1%) developed new lesions when the primary lesions progressed. After their occurrence, most of the newly developed lesions (32, 76.2%) were directly absorbed (**Figure 2**). On the latest CT, patients with new lesions had more complex residues than those without new lesions.

## Discussion

The present study revealed that the development of new pulmonary lesions during treatment was common, and was mainly related to lymphopenia, elevated CRP, or primary lesions progression, but irrelevant to the extent of primary lesions and other clinical characteristics. Previous studies have revealed that COVID-19 may be associated with cellular immune and acute severe systemic inflammatory responses 14, 15. Therefore, the development of new lesions indicated that primary lesions were more severe and could not be well-controlled by conventional treatment. Also, more complex residues on the latest CT in patients with progressed primary lesions also confirmed this finding. It might be because the population is susceptible to this disease, and there is no specific treatment at present, so patients mainly rely on autoimmunity 16, 17. Thus, the patients with worsening laboratory indicators should be monitored closely.

In this study, new lesions developed in the early stage of the disease, indicating that COVID-19 cannot be controlled quickly, and SARS-CoV-2 can be transmitted easily in the early stage, which was consistent with other results 4, 14, 18. The SARS-CoV-2 load usually peaked at the symptom onset, which was different from that of SARS-CoV and MERS-CoV 19, 20. The high viral load of SARS-CoV-2 in the early stage represents its high infectivity in this period, which could explain its extensive transmission. Over time, the viral load gradually declines, indicating that the infectivity gradually decreases during treatment. However, some patients in this study still developed new lesions up to 15 days after treatment. Thus, the disease's longer communicable period should be given enough attention. For patients with new lesions, their nucleic acid test turned negative after all new lesions appeared, which showed a good consistency between CT and nucleic acid test results. Therefore, these two diagnostic methods should be combined to determine the potential infectivity from macroscopic and microscopic views.

On CT images, newly developed lesions at any period were usually multiple and distributed not only in previously involved lobes but also in uninvolved lobes, which was consistent with SARS-CoV-2's strong infectivity. Thus, the rapid progression of pulmonary lesions attributed to both the development and dissemination of lesions. GGOs with or without consolidation were the main CT manifestations of COVID-19 pneumonia, which was also found in other studies 21-23. Regarding new lesions, they showed similar manifestations with primary lesions but had a relatively limited extent. Most of them were absorbed directly after occurrence, which suggested that the treatment efficiently decreased the virus' ability to damage lung tissue and controlled its progression.

This study has two limitations. First, the interval and number of follow-up CT scans in different patients were different, so the occurrence time of new lesions may be inaccurate. However, multiple follow-up CT scans were helpful for understanding the CT characteristics and evolution of the new lesions. Second, primary lesions in some patients were more extensive, which may cover or mix with new lesions during progression, thus making new lesions undetectable.

## Conclusions

Most patients with COVID-19 pneumonia would develop new pulmonary lesions during treatment, especially those with lymphopenia, elevated CRP, or primary lesion progression. These newly developed lesions usually manifest as multiple GGOs with or without consolidation, distribute around primary lesions or in the previously uninvolved lobes, and subsequently are absorbed directly. CT can be used along with nucleic acid tests to evaluate the outcomes of the disease and its infectivity.

## Figures and Tables

**Table 1:** The clinical and laboratory data of patients with COVID-19

	Patients with new lesions (n=42)	Patients without new lesions (n=14)	*P-*value
**Age**	49.6±16.1 (14-89)	44.1±13.0 (16-67)	0.253^&^
**Gender**			
Man	28 (66.7)	8 (57.0)	0.520^*^
Woman	14 (33.3)	6 (43.0)	
**Clinical type**			
Mild	2 (4.8)	0 (0)	
Moderate	34 (81.0)	13 (93.0)	0.811^**^
Severe	6 (14.3)	1 (7.0)	
**Clinical manifestations at admission**			
Fever	30 (71.4)	8 (57.0)	
Cough	28 (66.7)	10 (71.0)	
Sputum	8 (19.0)	3 (21.0)	
Dyspnea	4 (9.5)	3 (21.0)	
Diarrhea	4 (9.5)	2 (14.0)	
**Laboratory indicators**			
leucocyte (× 10^9^/L, 3.5-9.5)	4.9±2.1 (1.5-11.8)	5.9±1.8 (2.0-8.9)	
Decreased	8 (19.0)	1 (7.0)	
Normal	32 (76.2)	13 (93.0)	0.376^**^
Increased	2 (4.8)	0 (0)	
Lymphocyte(× 10^9^/L, 1.1-3.2)	1.0±0.5 (0.4-2.8)	1.2±0.5 (0.2-2.1)	
Decreased	28 (66.7)	5 (36.0)	0.041*
Normal	14 (33.3)	9 (64.0)	
CRP (mg/L, 0-8)	25.6±24.6 (0.5-94.9)	14.2±33.2 (0.5-124.1)	
Increased	33 (78.6)	2 (14.0)	0.000^*^
Normal	9 (21.4)	12 (86.0)	
LDH (U/L, 12-250)	535±173 (142-1024)	440±166 (177-860)	
Increased	39 (92.9)	13 (93.0)	1.000^*^
Normal	3 (7.1)	1 (7.0)	
PaO2 (kpa, 80-100)	91±28 (58-179)	109±37 (54-167)	
Decreased	15 (35.7)	4 (28.6)	
Normal	17 (40.5)	4 (28.6)	0.553^**^
Increased	10 (23.8)	6 (42.8)	
**Follow-up time (d)**	18±5 (10-31)	15±5 (10-29)	0.128^*^
**Numbers of CT scans**	4±1 (2-6)	4±1 (2-6)	0.294^#^

Notes: (a) Continuous data were presented as mean ± standard deviation (minimum-maximum), while the categorical data were presented as count (percentage of the total); (b) Abbreviations: COVID-19: coronavirus disease 2019; CRP: C-reactive protein; LDH: lactate dehydrogenase; PaO_2_: partial pressure of oxygen; (c) *Pearson χ^2^ test; ^**^ Fisher exact test; ^#^ Wilcoxon rank sum test; ^&^ Analysis of variance.

**Table 2:** The CT features of new lesions in patients with COVID-19

Characteristics	Patients with new lesions (n=42)
**Interval of initial CT to the first repeat CT with new lesions**	
1-4 day	23 (54.8)
5-8 day	8 (19.0)
9-12 day	8 (19.0)
13-15 day	3 (7.1)
Number	
Multiple	38 (90.5)
Solidary	4 (9.5)
1-4 day	6.3±4.8(1-20)
5-8 day	7.6±5.8 (1-18)
9-12 day	6.1±4.8 (1-15)
13-15day	3.2±3.2 (1-9)
**Distribution**	
Previously involved lobes	15 (35.7)
Previously uninvolved lobes	3 (7.1)
Both	24 (57.1)
**Density**	
Pure GGOs	19 (45.2)
GGOs with consolidation	23 (54.8)
**Shape**	
Small patchy	39 (92.9)
Nodular	14 (33.3)
Both	11(26.2)

Note: Continuous data were presented as mean ± standard deviation (minimum-maximum), while the categorical data were presented as count (percentage of the total); Abbreviations: COVID-19: coronavirus disease 2019, GGOs: ground-glass opacities.

**Table 3:** Follow-up CT characteristics of COVID-19 patients with/without new lesions

	Patients with new lesions (n=42)	Patients without new lesions (n=14)	*P-*value
**Follow-up time**			
Mean ± SD (range)	18±5 (10-31)	15±5 (10-29)	0.128^&^
**Interval between initial CT and first negative nucleic acid test**			
Mean ± SD (range)	18±8 (5-40)	15±7 (5-40)	0.163^#^
**Changes of primary lesions**			
Absorption after progression	38 (90.5)	2 (14.3)	0.000^**^
Direct absorption	4 (9.5)	12 (85.7)	
**Changes of new lesions**			
Absorption after progression	10 (23.8)	-	
Direct absorption	32 (76.2)	-	
**CT manifestations on latest CT**			
Pure GGOs	9 (21.4)	9 (64.3)	0.006^**^
GGOs with fibrous stripes/ consolidation	33 (78.6)	5 (35.7)	

Note: Continuous data were presented as mean ± standard deviation (minimum-maximum), while the categorical data were presented as count (percentage of the total); * Pearson χ^2^ test; ** Fisher exact test; ^#^ Wilcoxon rank sum test; ^&^ Analysis of variance; Abbreviations: COVID-19: coronavirus disease 2019, GGOs: ground-glass opacities.

## Abbreviations

COVID-19: coronavirus disease 2019
SARS-CoV-2: severe acute respiratory syndrome coronavirus 2
GGOs: ground-glass opacities
PCR: polymerase chain reaction
CRP: C-reactive protein
LDH: lactate dehydrogenase
PaO_2_: partial pressure of oxygen
